# The association between Darier’s disease and schizophrenia : a case report

**DOI:** 10.1192/j.eurpsy.2023.1602

**Published:** 2023-07-19

**Authors:** M. Abdelkefi, F. Guermazi, N. Bouattour, F. Cherif, I. Baati, J. Masmoudi

**Affiliations:** psychiatry A, Hedi Chaker university hospital, sfax, Tunisia

## Abstract

**Introduction:**

Darier’s disease, also known as Darier-White disease or keratosis follicularis, is a rare autosomal dominant genodermatosis. Clinical experience has long suggested an association between neuropsychiatric abnormalities and Darier’s disease. Moreover, associations with mental retardation, schizophrenia, mood disorders and suicide have been reported.

**Objectives:**

We studied the association between Darier’s disease and schizophrenia.

**Methods:**

We illustrate a case of schizophrenia and Darier’s disease comorbidity with a small review of the literature that summarizes the characteristics of such an association.

**Results:**

Mrs SD, 48 years old, with a prior history of schizophrenia, moderate intellectual disability and several hospitalizations in psychiatry.

She was hospitalized in our department of psychiatry "A" of the Hedi Chaker university hospital after she was brought by the police for odd and disorganized behavior, environmental violence and refusal of treatment.

On somatic examination, the presence of crusty maculopapular skin lesions, non-pruritic, yellowish brown in color and a few millimeters in diameter, located on the back of both hands and feet, face and neck was noted. The patient reported that her brother has similar skin lesions. A dermatological consultation was sought for assessment of her skin condition and a skin biopsy confirmed the diagnosis of Darier’s disease.

**Conclusions:**

Schizophrenia and intellectual disabilities are frequently associated with Darier’s disease. Physicians should be aware of this association in order to allow a rapid diagnosis and early management of psychiatric disorders associated with this genodermatosis.

**Disclosure of Interest:**

None Declared

